# Scaling up production of cephalosporin C by *Acremonium chrysogenum* W42-I in a fermenter using submerged fermentation

**DOI:** 10.1186/s13568-024-01778-1

**Published:** 2024-11-05

**Authors:** Asmaa A. Ibrahim, Ghadir S. El-Housseiny, Khaled M. Aboshanab, Ansgar Stratmann, Mahmoud A. Yassien, Nadia A. Hassouna

**Affiliations:** 1 Department of Microbiology and Immunology, Faculty of Pharmacy, Ain Shams University, Organization of African Unity St., POB: 11566, Abbassia, Cairo, 11566 Egypt; 2 W42 Industrial Biotechnology GmbH, 44227 Dortmund, Germany

**Keywords:** Cephalosporin C, Scaling up, Fermentor, *Acremonium chrysogenum*

## Abstract

Cephalosporins presently stand as the most extensively utilized antibiotic in clinical settings. *Acremonium (A.) chrysogenum* is the main strain used in the manufacturing of cephalosporin C (CPC), which offers distinct advantages, including a wide-ranging antibacterial spectrum and powerful antibacterial efficacy. Our study aimed to determine the optimal conditions for scaling up the production of CPC from *A. chrysogenum* W42-I starting with the optimized conditions on the shake flask level obtained from our previous study and utilizing the optimized media (CPC2). The results indicated that an inoculum size equivalent to 1% v/v, aeration at 1 vvm, and an agitation rate of 400 rpm, with controlled pH at 4, were the most favorable conditions for the CPC production using a laboratory fermentor (14 L). The concentration of generated CPC was assessed using two standard curves obtained from agar well diffusion and high-performance liquid chromatography (HPLC). These optimized conditions resulted in a production of 399.52 µg/mL showing a significant increase of approximately 3.4 folds when compared to the unoptimized fermentation run. In conclusion, our findings demonstrated a more favorable time course for CPC production in the fermentor compared to that in the shake flask. Notably, there was a two-fold increase in production within the first three days. Fortunately, the fermentor achieved a noteworthy increase in output, generating 1.598 gm of the CPC within 4 L.

**Supplementary Information:**

The online version contains supplementary material available at 10.1186/s13568-024-01778-1.

## Key points

- Cephalosporin C production was successfully scaled up and optimized using a 14 L fermentor resulting in about 3.4 folds as compared to the unoptimized condition.
- Using the lab fermentor (14 L), a two-fold increase of CPC was attained in the first three days as compared to that of the shake flask.
- Using the lab fermentor (14 L), the fermentor achieved a noteworthy increase in output, generating 1.598 gm of the CPC/4 L fermentation medium.

**Supplementary Information:**

The online version contains supplementary material available at 10.1186/s13568-024-01778-1.

## Introduction

*Acremonium chrysogenum*, categorized among filamentous fungi (Tian et al. 2017b), holds significant importance in industrial applications (Liu et al. 2022). One of its metabolites, cephalosporin C (CPC), serves as a primary source for the synthesis of 7-amino cephalosporanic acid (7-ACA), a fundamental intermediate for the production of several frontline anti-infectious cephalosporin antibiotics in the industrial sector (Lin and Kück 2022). Given the heightened sensitivity of filamentous fungi to environmental variations, gaining insights into the distinctive physiological and metabolic traits of a specific strain becomes instrumental in devising favorable culture conditions that promote optimal growth and production (Bartoshevich et al. 1990). The industrialization of CPC fermentation was achieved through breakthroughs in key technologies encompassing fermentation yield, regulation, and preparation and purification, as documented in previous studies (Adinarayana et al. 2003; Cruz et al. 2004; Ahsan et al. 2017; Liu et al. 2022).

To meet the demand for substantial quantities of semi-synthetic cephalosporin, it becomes imperative to produce the key intermediate (CPC) in large volumes through cost-effective and efficient production routes. Fermentors offer comprehensive monitoring and control capabilities. Sensors and automation systems allow real-time tracking of crucial parameters, enabling precise control over the fermentation process for consistent results (Duan et al. 2012, 2014). The challenges associated with poor fermentation performance on a large scale are often considered a top-priority risk during scale-up due to the intricate nature of the fermentation process. Numerous parameters influence performance, and a majority of these factors are susceptible to changes throughout the scale-up process (Crater and Lievense 2018). The objective of the present research was to scale up the production of CPC from *A. chrysogenum* W42-I, whose production was optimized on the shake flask level in our previous study (Ibrahim et al. 2023). This involved exploring various environmental factors during the fermentation process, including pH, dissolved oxygen levels, agitation, and inoculum size.

## Materials and methods

### Microorganisms

*Acremonium chrysogenum* W42-I (W42 GmbH, strain collection) (https://www.w42biotechnology.de/) was deposited in the Culture Collection Ain Shams University (CCASU) under the accession code CCASU-2020-W42-I (Ibrahim et al. 2023). The strain was stored as previously reported (Hopwood and Wright 1978; Kieser et al. 2000). *Staphylococcus aureus* ATCC 25,923 was well-maintained in Luria–Bertani (LB) broth enriched with 50% glycerol and preserved at -20 °C (Miller 1983). In order to evaluate the production of CPC through the implementation of the agar well diffusion method, the strain was cultivated on Mueller Hinton agar at a temperature of 37 °C for a duration of 24 h. (Balouiri et al. 2016).

### Inoculum

To generate a spore suspension of *A. chrysogenum* W42-I, a culture that was three days old on slants was vigorously agitated for a duration of one minute with a sterile solution of saline (0.9% NaCl). The count of the spores was measured using a haemocytometer, employing the procedure outlined by Liu (2016) and adjusted to around 10^8^ spores per milliliter (Lotfy 2007). This prepared spore suspension was utilized as the seed culture.

### Culture media

The basal medium used for *A. chrysogenum* W42-I was CPC2 medium which was mentioned in our previous study (Ibrahim et al. 2023).

### CPC production in shake flask

CPC production in a shake flask was conducted to compare its production with that using a fermentor (CelliGen 310, Eppendorf AG, Hamburg, Germany). The experimental conditions employed were the ones achieved through optimization in our previous investigation (Ibrahim et al. 2023). In brief, 25 mL of CPC2 media were inoculated with 1% v/v of the spore suspension prepared (10^8^ spores/mL) and incubated at 200 rpm, initial pH of 4 and 28 °C. At specific time intervals, samples were collected from the culture broth to extract and determine the concentration of CPC.

### CPC production in fermentor

Cephalosporin C (CPC) production by *A. chrysogenum* W42-I was investigated in a 14 L laboratory glass fermentor operating in batch mode with a 4 L working volume. pH control, when needed, utilized sterile 3 M NaOH and 3 M HCl. The production medium and necessary chemicals underwent sterilization by autoclaving. Temperature was regulated with a Resistance Temperature Detector (RTD) and a cooling water system. Agitation employed a Rushton turbine impeller, and sterile air, filtered and compressed, entered the vessel through a 0.22 μm cartridge filter. Dissolved oxygen percentage (DO%) and pH were monitored using sensors, and exhaust gases passed through a water-cooled condenser before returning to the vessel. During all batch fermentation processes, the CPC2 medium served as the production medium, and the temperature was upheld at 28 °C. Throughout each experiment, samples were extracted at diverse time intervals to monitor the production of CPC.

### Studying environmental parameters affecting CPC production

Several experiments were conducted to investigate various factors influencing CPC production by the examined strain. These factors encompassed the time course of CPC production, inoculum size, aeration rate, pH, and agitation rate. Unless otherwise mentioned all experiments were done in triplicate and the mean value was calculated.

### Studying the time course of CPC production

The experiment (Run 1) was carried out using 3.96 L of CPC2 medium and inoculated with seed culture at 1% v/v (40 mL) to obtain a final volume of 4 L. The initial pH was adjusted at 4 before sterilization and no significant change occurred after sterilization. The agitation rate was maintained at 200 rpm and sterile air was introduced into the vessel at a constant flow rate of 0.5 vvm. Samples were withdrawn from the fermentor at specific time intervals for a period of 6 days for monitoring CPC production.

### Effect of aeration rate

The influence of the aeration rate was tested at two rates of 1 vvm (Run 2) and 2 vvm (Run 3) under the same condition in Run 1. The obtained results were compared to that of Run 1 (0.5 vvm).

### Effect of agitation rate

The effect of agitation was studied at two different agitation speeds of 300 rpm (Run 4) and 400 rpm (Run 5). In both cases, the conditions used were inoculum size 1% v/v, 1 vvm and initial pH 4 (uncontrolled). The results were compared to those obtained in Run 2 (using the same conditions but 200 rpm).

### Effect of inoculum size

The CPC production was tested using the same conditions of Run 5 except at 2 different inoculum sizes of 2.5% v/v (Run 6) and 5% v/v (Run 7) and the results were compared to those obtained in Run 5 (using inoculum size 1% v/v).

### Effect of pH

CPC production was tested using controlled pH 4 (Run 8), the conditions applied were the same as those used for Run 5. The results were compared to those obtained in Run 5 (using uncontrolled pH 4).

### Evaluation of the antibacterial activity

The antibacterial activity of the CPC produced by this strain was evaluated against *S. aureus* ATCC 25,923 using the agar well diffusion method as previously described by Balouiri et al. (2016). The plates were cooled at 4–8 ºC for a minimum of 30 min to enhance the spread of the culture filtrate. Subsequently, after a 24-hour incubation period at 37 °C, the diameters of the inhibition zones were measured.

### Evaluation of the produced CPC

Different concentrations of the standard CPC (Sigma-Aldrich, Saint Louis, MO, USA) were drawn against the mean inhibition zone diameters to construct the standard curve (Fig. S1; Supplementary file). The proposed linear Eq. (1) used to calculate the CPC concentrations was as follows: y = 13.05x + 29.2.

y is the mean inhibition zone (mm) with R² = 0.9646; x is log the CPC concentration in mg/mL.

### Reverse phase high-performance liquid chromatography (HPLC) analysis

Reverse phase HPLC was utilized to estimate the CPC concentration versus Area Under the Curve (AUC). The procedure involved a RP-C18-UPLC-shimpack 2 mm x 150 mm particle size 1.7 μm column, operating at a temperature of 30 °C and a UV detector set at 260 nm. The mobile phase, moving at a speed of 0.2 mL/min, consists of solvent A, which is a mixture of 0.1% formic acid in water, and solvent B, which is a blend of 0.1% formic acid in acetonitrile. Before analysis, 500 µL samples underwent centrifugation (10 min at 10,000 xg) and were then diluted 1:10 with the mobile phase (Ibrahim et al. 2023). Standard CPC solutions (50 mg/L) were prepared in the mobile phase, filtered (0.45 μm) and 10 µL was injected for a duration time of 10 min. The recommendation included the preparation and storage at -20 °C of a standard CPC solution (1 g/L) in the mobile phase.

**Fig. 1:**
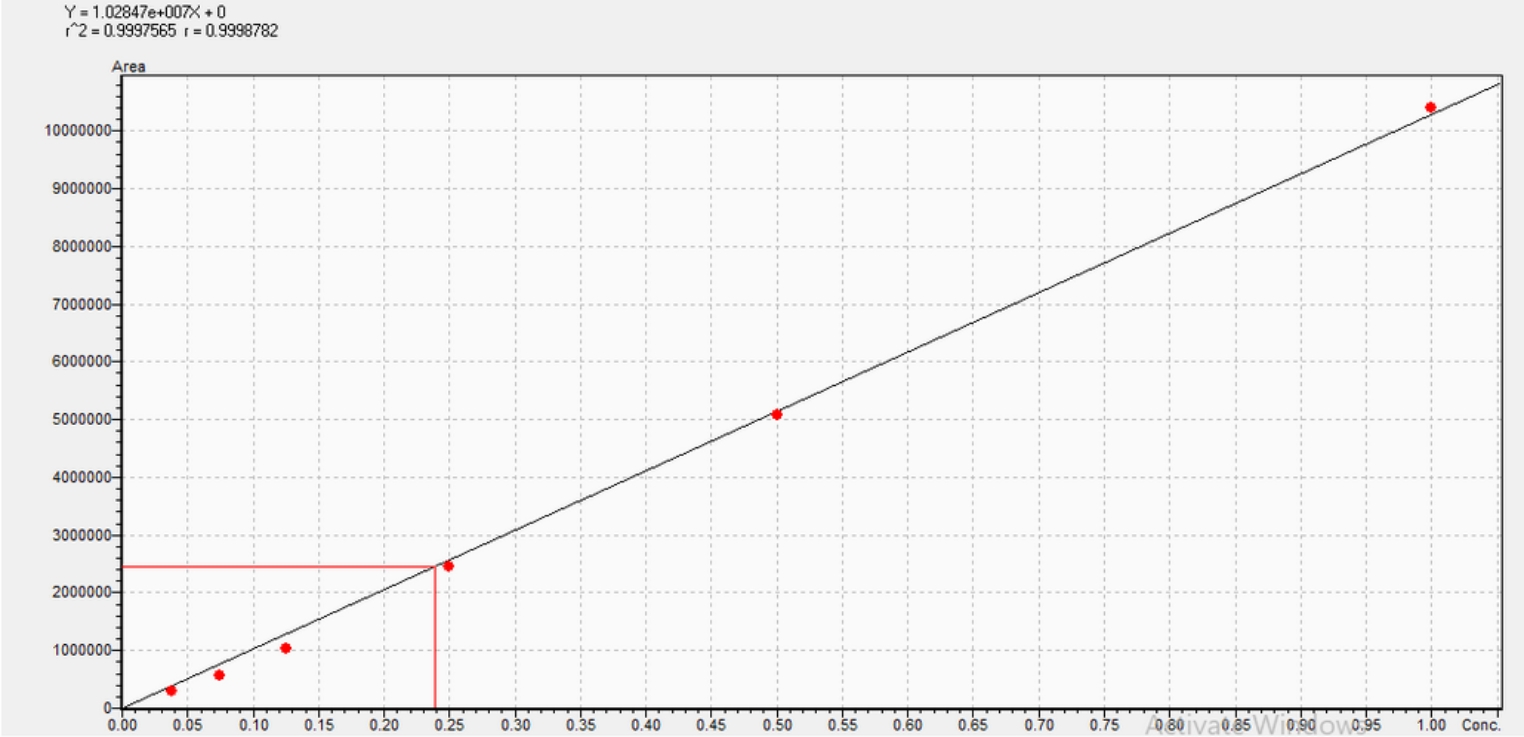
Standard Curve of standard CPC concentration vs. corresponding Area Under the Curve (AUC)

The CPC concentrations were drawn versus the Area Under the Curve (AUC) and a standard curve was constructed (Fig. 1). The proposed linear Eq. (2) was employed to calculate a CPC concentration was as follows:

$${\text{y}}=1.02847\times10^7{\text{x}}+0$$

y is the AUC with R² = 0.9997; x is CPC concentration in mg/mL.

## Results

### CPC production in shake flask

After 4 days of incubation using optimized media and conditions in shake flask fermentation, the maximum CPC concentration reached 424.2 µg/mL.

### CPC production in laboratory fermentor

#### Time course of CPC production

**Fig. 2:**
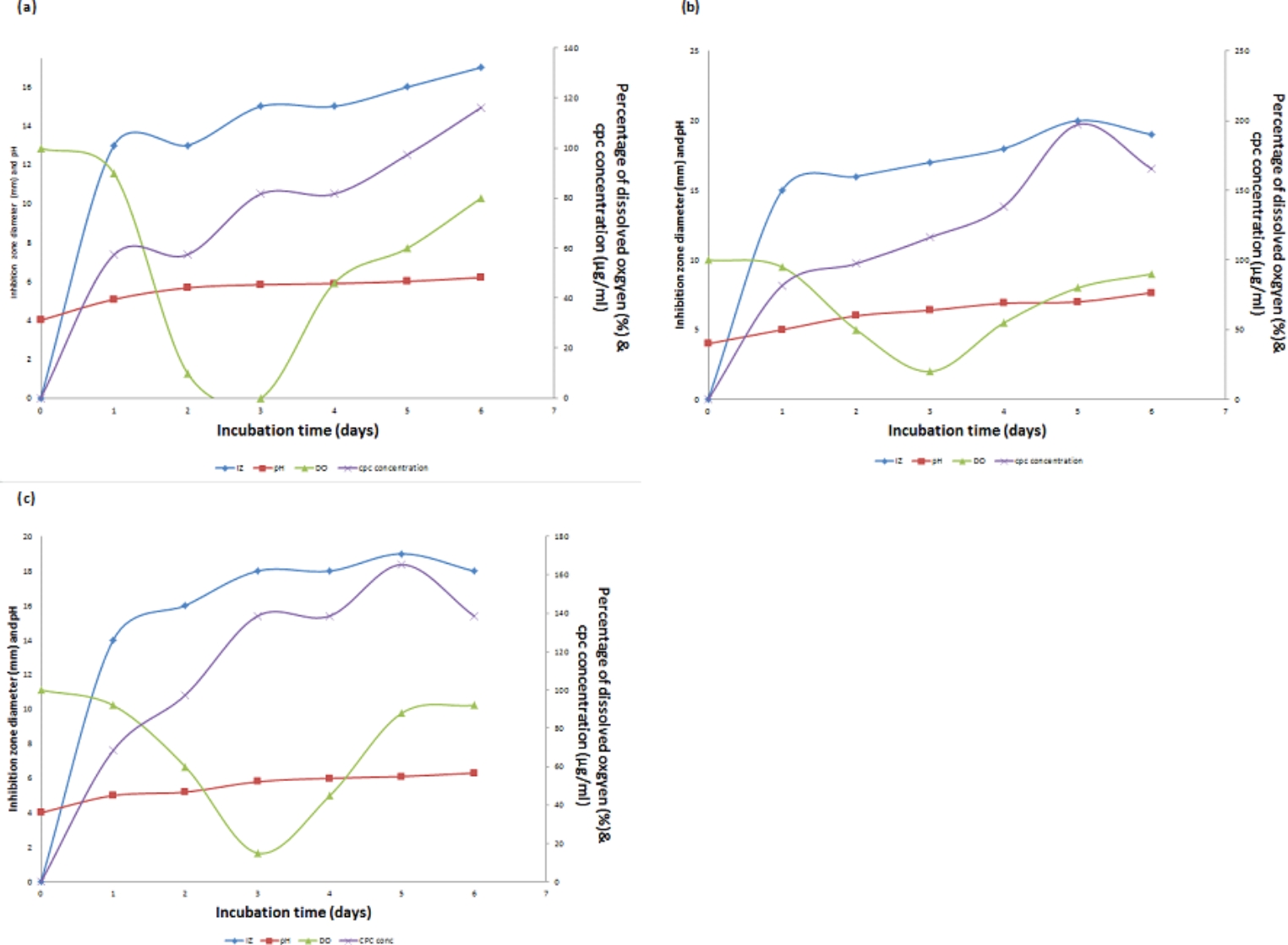
**(a)** Time course of antibiotic production, concentration, pH and DO% for CPC production by *A. chrysogenum* W42-I in a laboratory fermentor using batch fermentation mode (Run 1). Applied conditions: Inoculum size of 1% v/v; uncontrolled initial pH of 4; temperature of 28 °C; agitation rate of 200 rpm and aeration rate of 0.5 vvm. **(b)** Time course of antibiotic production, concentration, pH and DO% for CPC production by *A. chrysogenum* W42-I in a laboratory fermentor using batch fermentation mode (Run 2). Applied conditions: Inoculum size of 1% v/v; uncontrolled initial pH of 4; temperature of 28 °C; agitation rate of 200 rpm and aeration rate of 1 vvm. **(c).** Time course of antibiotic production, concentration, pH and DO% for CPC production by *A. chrysogenum* W42-I in a laboratory fermentor using batch fermentation mode (Run 3). Applied conditions: Inoculum size of 1% v/v; uncontrolled initial pH of 4; temperature of 28 °C; agitation rate of 200 rpm and aeration rate of 2 vvm

Time course profile of CPC production is shown in Fig. 2a (Run 1). As shown in the Figure, CPC concentration increased gradually to reach a maximum value of 116.18 µg/mL at day 6. Concerning DO, it decreased rapidly to reach its lowest point of 0% after about 72 h. The DO then increased again rapidly to 46% at day 4 then slowly rose to reach 80% at the end of the run. On the other hand, pH increased slightly to reach 6.2 at the end of the fermentation.

### CPC production using different aeration rates

Two runs were done with the same conditions listed before (run 1) but at two different aeration rates (1 vvm or 2vvm). In case of using 1 vvm (Run 2) and 2 vvm (run 3), as shown in Fig. 2b & c, CPC production started after 24 h of fermentation, but occurred with an increased production rate to reach a maximum of 197.25 and 165.35 µg/mL, respectively at day 5 of the fermentation process.

**Fig. 3:**
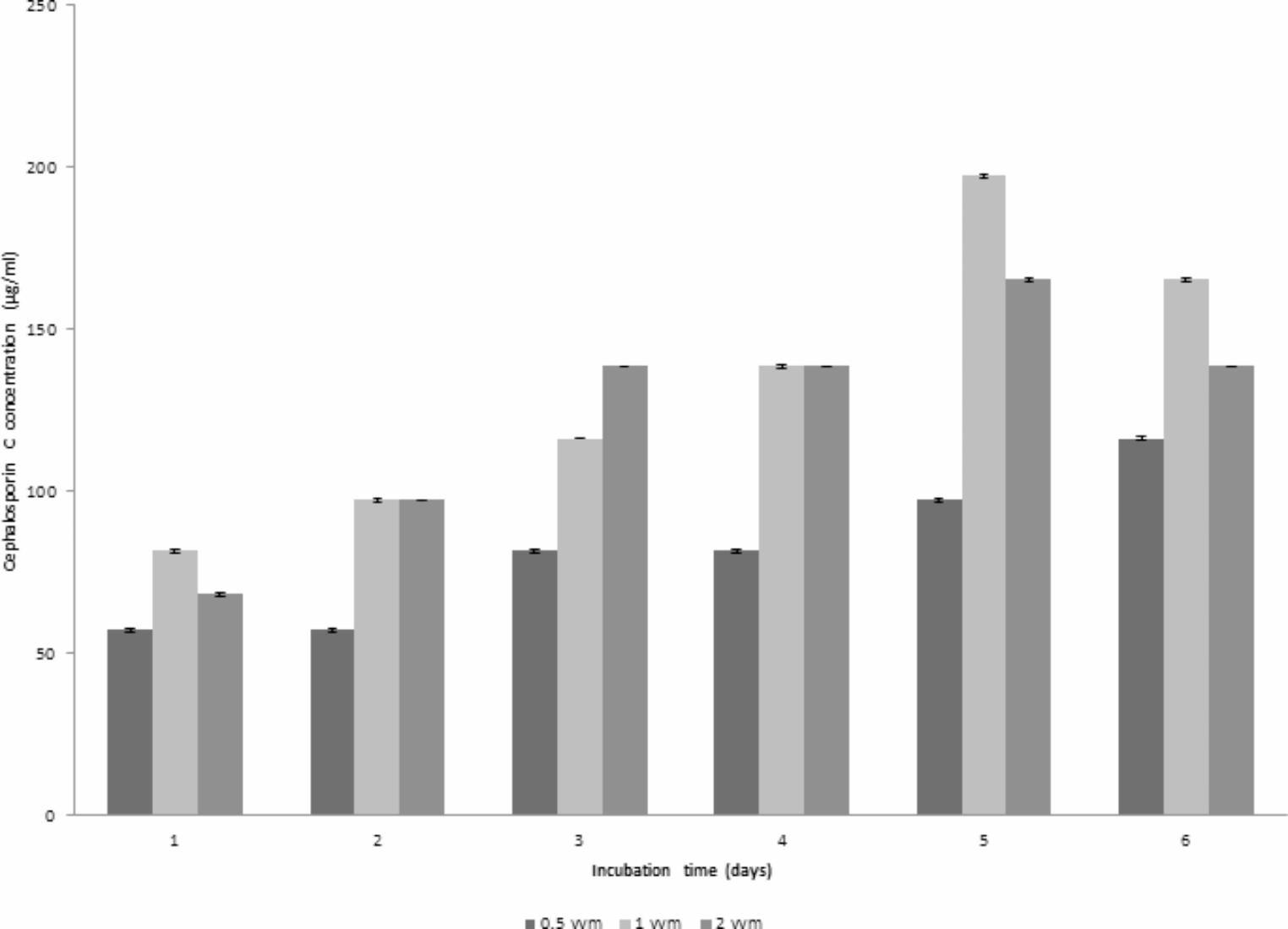
Comparison of maximum CPC production by *A. chrysogenum* W42-I in a laboratory fermentor at different aeration rates after 6 days of fermentation. Conditions applied: uncontrolled initial pH of 4; temperature of 28 °C; inoculum size of 1%v/v and agitation rate of 200 rpm

The DO profile using 1 vvm inoculum was similar to that obtained using 2 vvm where both reached their lowest point after 72 h, then elevated again to reach around 90% at the end of the run. Concerning pH, for using 2 vvm a profile similar to that obtained using 0.5 vvm was observed however a different pH course was obtained when using 1 vvm, where an increase in pH occurred to reach 7.66 at the end of fermentation. Taken together, an aeration level of 1 vvm was selected as the optimum level since it led to the highest maximum CPC production as shown in Fig. 3.

### CPC production using different agitation rates

**Fig. 4:**
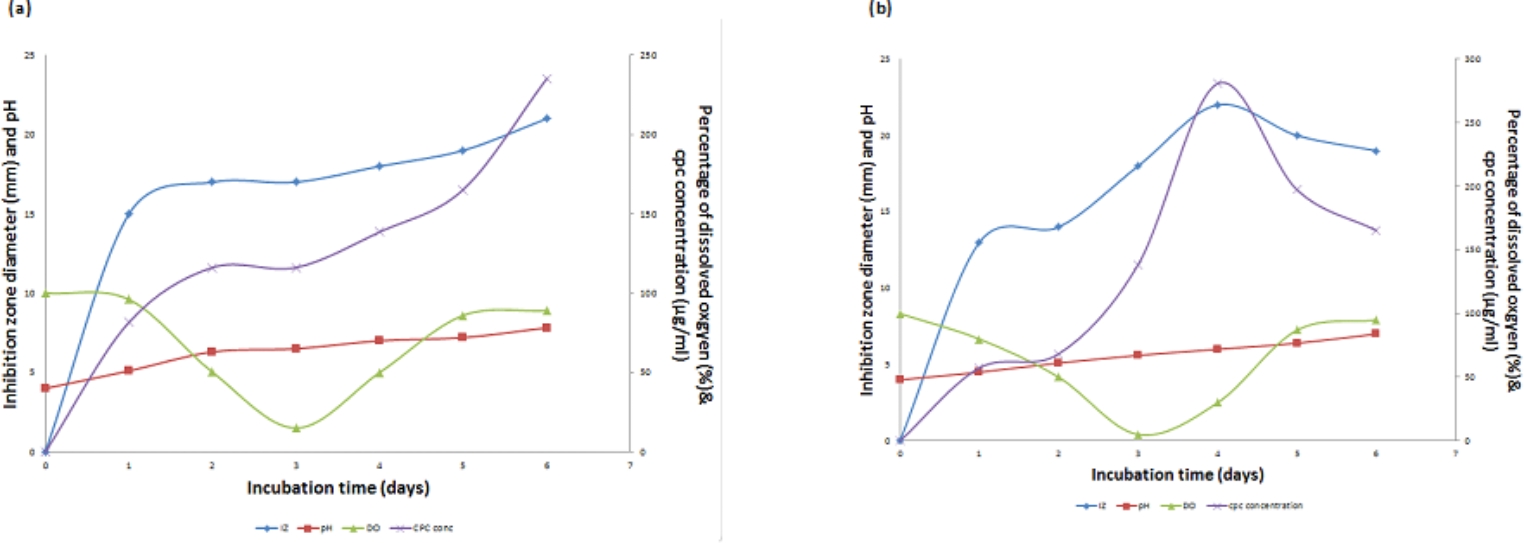
**(a)** Time course of antibiotic production, concentration, pH and DO% for CPC production by *A. chrysogenum* W42-I in a laboratory fermentor using batch fermentation mode (Run 4). Applied conditions: Inoculum size of 1% v/v; uncontrolled initial pH of 4; temperature of 28 °C; agitation rate of 300 rpm and aeration rate of 1 vvm. **(b)** Time course of antibiotic production, concentration, pH and DO% for CPC production by *A. chrysogenum* W42-I in a laboratory fermentor using batch fermentation mode (Run 5). Applied conditions: Inoculum size of 1% v/v; uncontrolled initial pH of 4; temperature of 28 °C; agitation rate of 400 rpm and aeration rate of 1 vvm

The effect of different agitation rates (300 and 400 rpm) on CPC production using an inoculum size of 1% v/v, temperature of 28 °C, aeration level of 1 vvm and initial pH of 4 was studied. As shown in Fig. 4a, maximum CPC level of 235.31 µg/mL was achieved using an agitation speed of 300 rpm (run 4) after 6 days of fermentation. The dissolved oxygen (DO) remained at elevated levels throughout the run, reaching a minimum of 15% after 3 days. Subsequently, DO levels experienced a resurgence, ultimately reaching approximately 89% by the end of the run. Conversely, the pH profile closely resembled that of the preceding run (Run 2), exhibiting an increase and nearly attaining a value of 7.82.

**Fig. 5:**
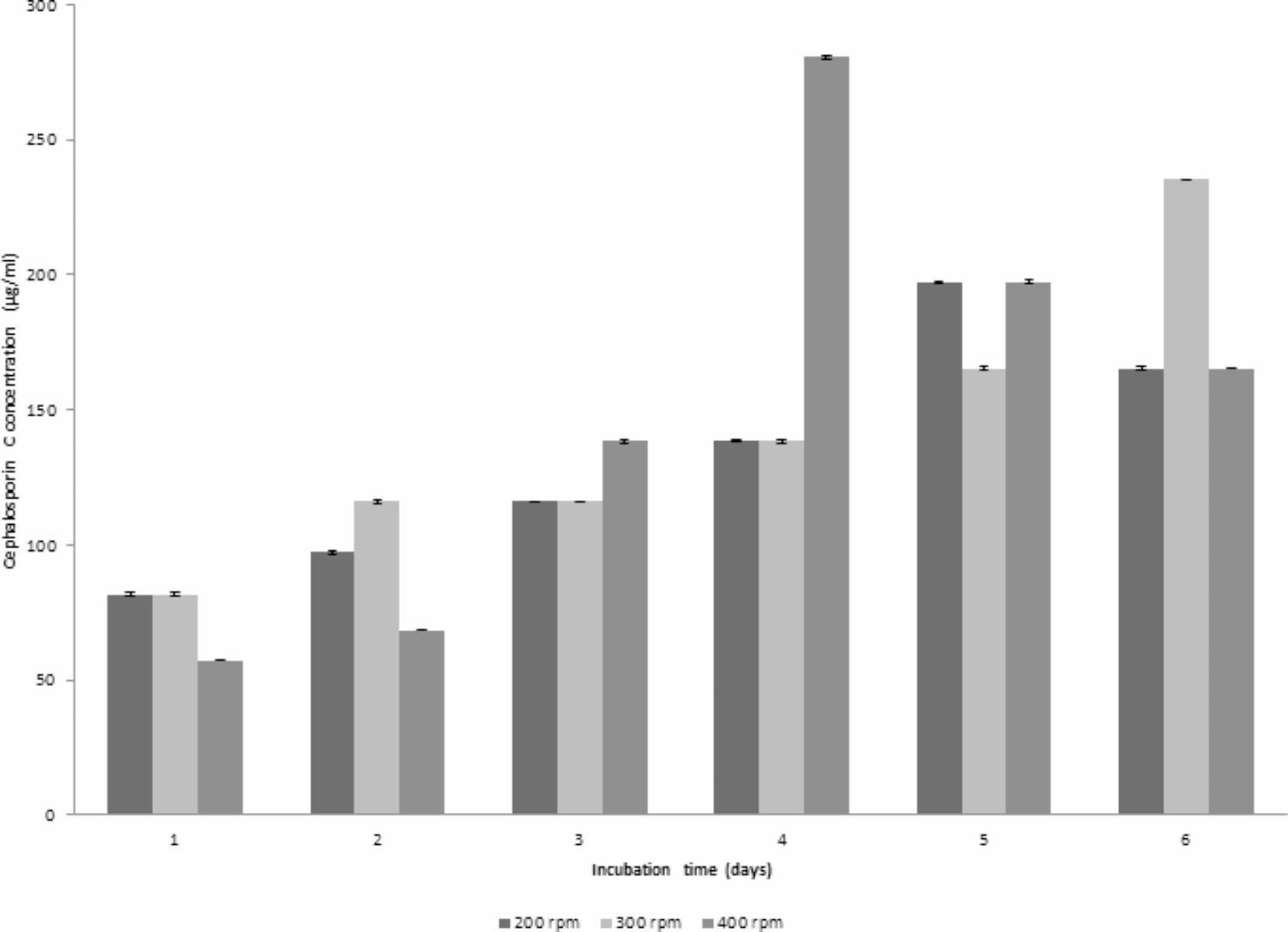
Comparison of maximum CPC production by *A. chrysogenum* W42-I in a laboratory fermentor at different agitation rates after 6 days of fermentation. Conditions applied: uncontrolled initial pH of 4; temperature of 28 °C; inoculum size of 1%v/v and aeration rate of 1vvm

At 400 rpm (Run 5), maximum CPC level of 280.72 µg/mL was accomplished after 4 days of fermentation. The dissolved oxygen (DO) consistently stayed elevated, reaching a minimum of 5% after 3 days and then recovering to around 95% by the end of the run. In contrast, the pH profile gradually increased, reaching a final value of 7 as illustrated in Fig. 4b. Accordingly, the optimum agitation rate was found to be 400 rpm since it led to maximum CPC production (Fig. 5).

### CPC production using different inoculum size

**Fig. 6:**
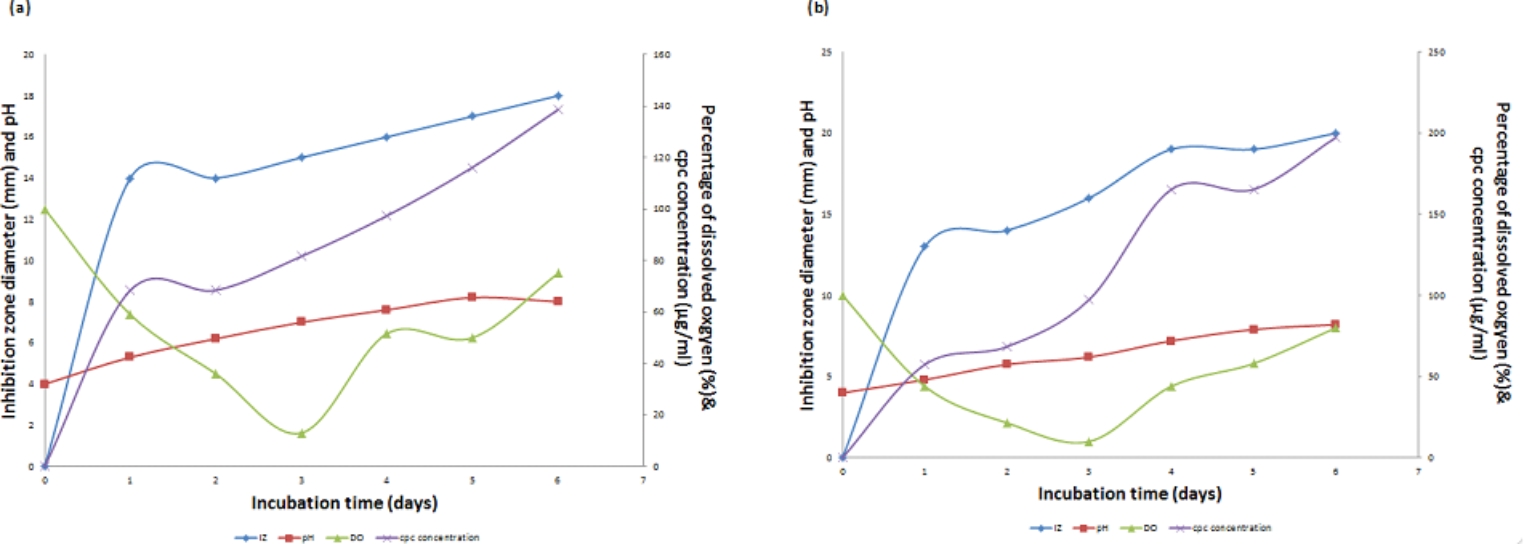
**(a)** Time course of antibiotic production, concentration, pH and DO% for CPC production by *A. chrysogenum* W42-I in a laboratory fermentor using batch fermentation mode (Run 6). Applied conditions: Inoculum size of 2.5% v/v; uncontrolled initial pH of 4; temperature of 28 °C; agitation rate of 400 rpm and aeration rate of 1 vvm.**(b)** Time course of antibiotic production, concentration, pH and DO% for CPC production by *A. chrysogenum* W42-I in a laboratory fermentor using batch fermentation mode (Run 7). Applied conditions: Inoculum size of 5% v/v; uncontrolled initial pH of 4; temperature of 28 °C; agitation rate of 400 rpm and aeration rate of 1 vvm

**Fig. 7:**
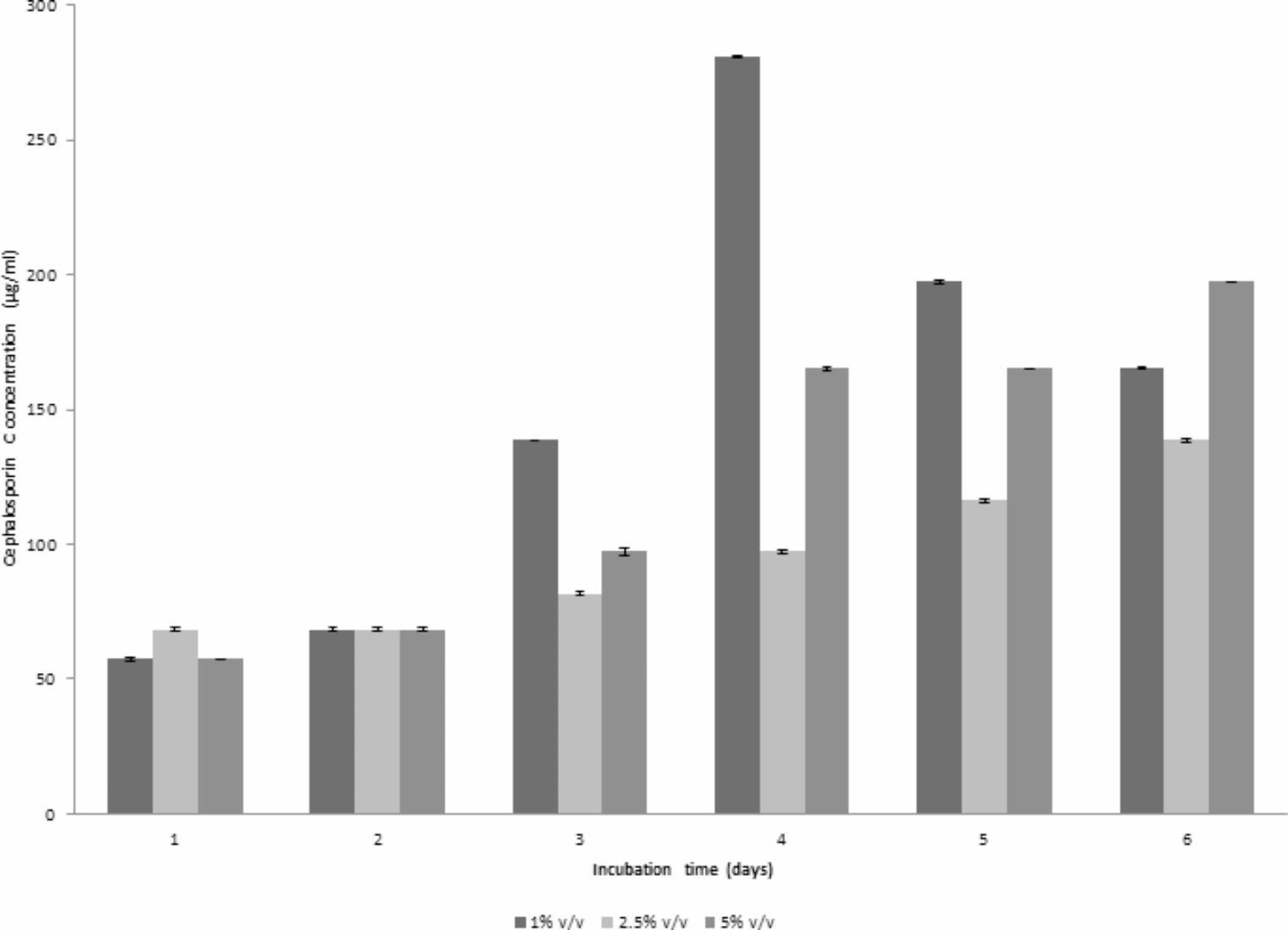
Comparison of maximum CPC production by *A. chrysogenum* W42-I in a laboratory fermentor at different inoculum sizes after 6 days of fermentation. Conditions applied: uncontrolled initial pH of 4; temperature of 28 °C; agitation rate of 400 rpm and aeration rate of 1 vvm

Two runs were carried out with the same conditions listed before (Run 5) but at two different inoculum sizes (2.5% or 5% v/v). In case of using 2.5% v/v (Run 6) and 5% v/v inoculum (Run 7), CPC production started after 24 h of fermentation, but occurred with an increased production rate to reach a maximum of 138.6 and 197.251 µg/mL, respectively, at day 6 of fermentation. The DO profile using 2% v/v inoculum was similar to that obtained using 5% v/v, both reaching their lowest point after 3 days, then elevating again to 75% for 2.5% v/v and 80% for 5% v/v at the end of the run. Concerning pH, the 2.5% v/v inoculum profile was similar to that obtained using 5% v/v inoculum. These findings were shown in Fig. 6a & b, respectively. Therefore, it can be concluded from Fig. 7 that an inoculum size of 1% v/v led to the highest maximum CPC production when compared to others. Therefore, this inoculum size was used in further studies.

### CPC production using controlled pH

**Fig. 8:**
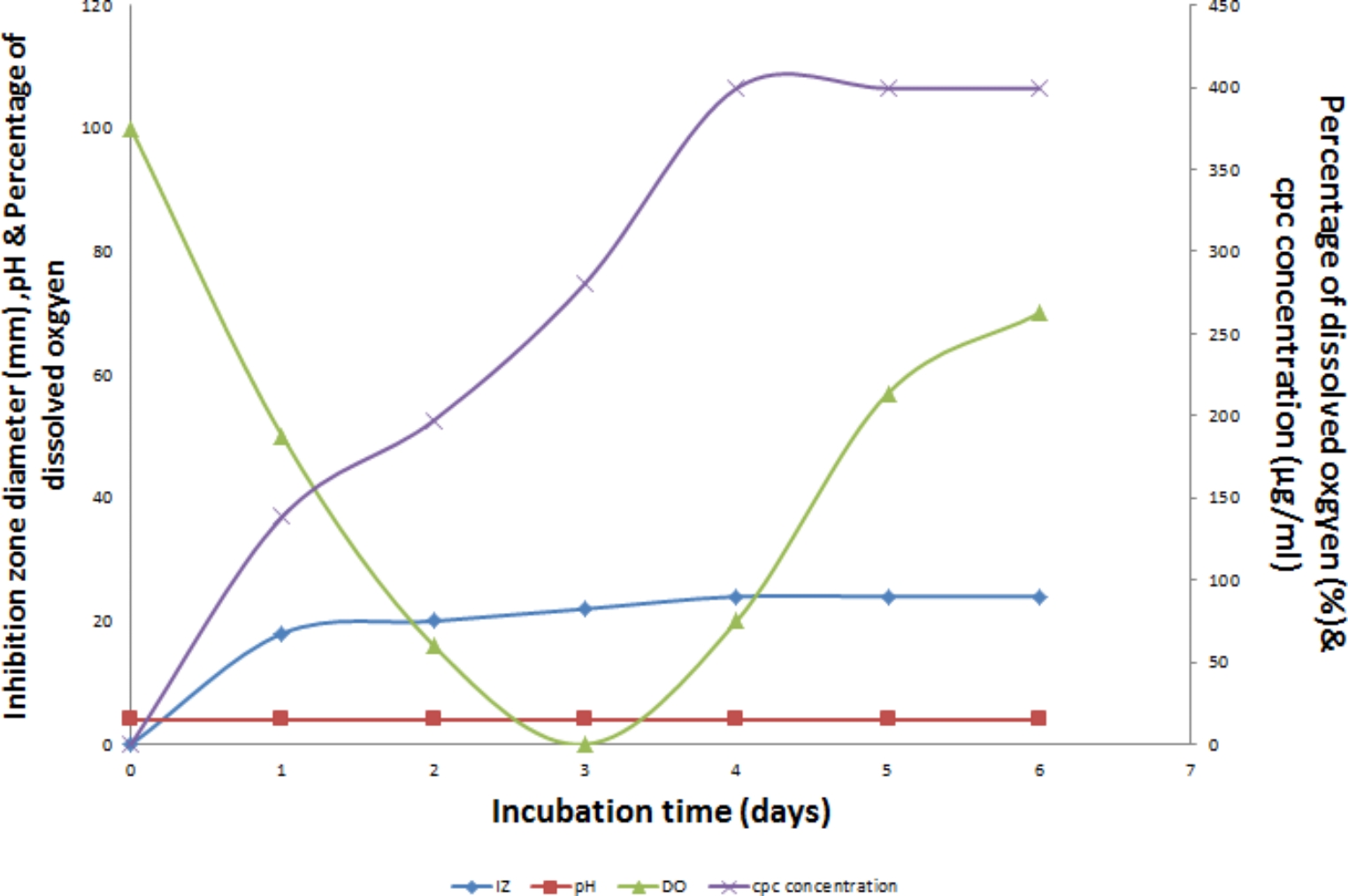
Time course of antibiotic production, concentration, pH and DO% for CPC production by *A. chrysogenum* W42-I in a laboratory fermentor using batch fermentation mode (Run 8). Applied conditions: Inoculum size of 1% v/v; controlled pH of 4; temperature of 28 °C; agitation rate of 400 rpm and aeration rate of 1 vvm

**Fig. 9:**
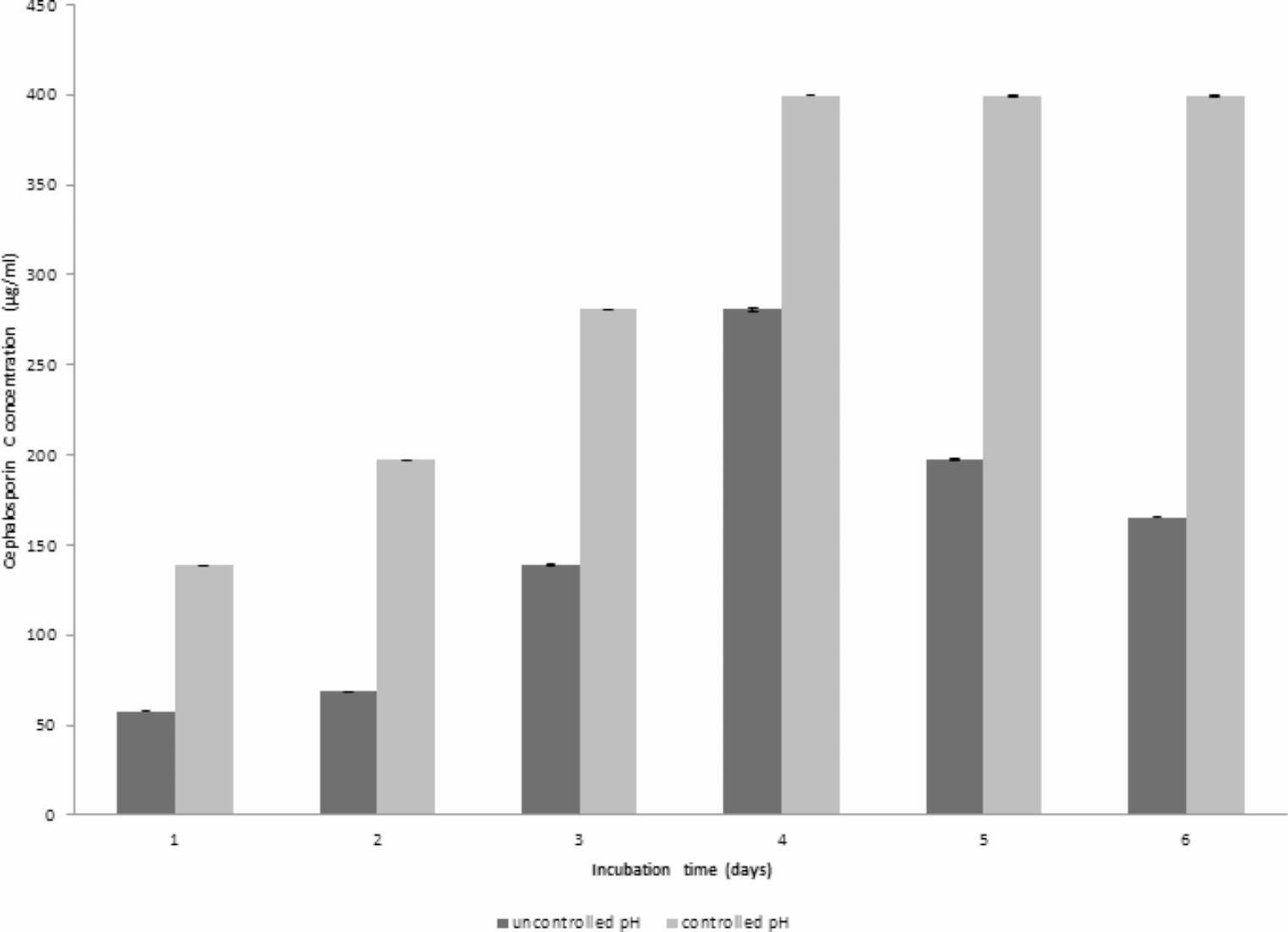
Comparison of maximum CPC production by *A. chrysogenum* W42-I in a laboratory fermentor using controlled vs. uncontrolled pH after 6 days of fermentation. Conditions applied: temperature of 28 °C; inoculum size of 1%v/v; aeration rate of 1 vvm and agitation rate of 400 rpm

**Fig. 10:**
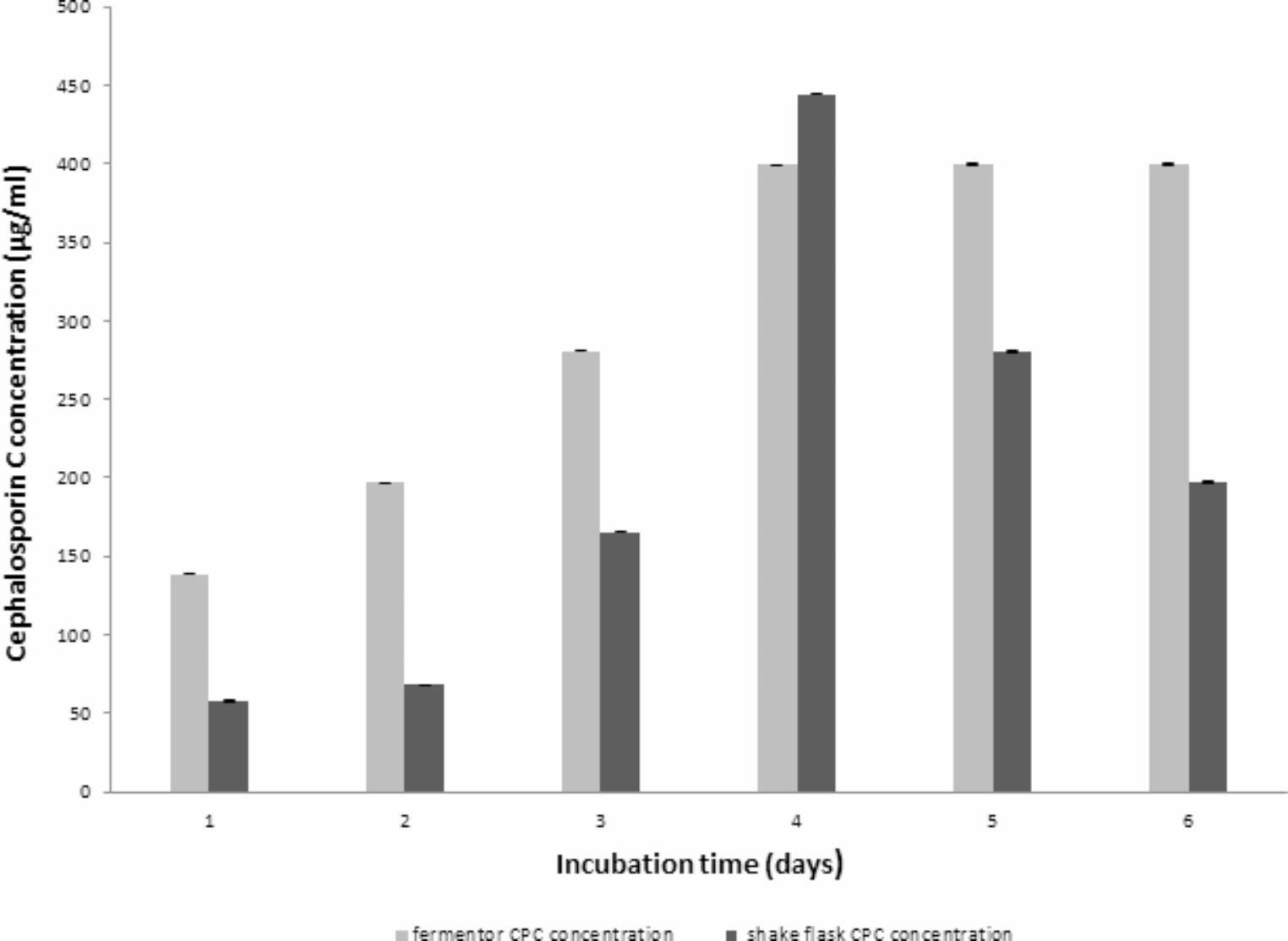
Comparing Maximum CPC production by *A. chrysogenum* W42-I in a laboratory fermentor vs. shake flask

The results presented in Fig. 8 indicate a noteworthy impact of controlled pH at 4 (Run 8) on the maximum production of CPC, reaching 399.52 µg/mL after 4 days of fermentation. This is in contrast to uncontrolled pH conditions where the maximum CPC concentration was 280.722 µg/mL, as depicted in Fig. 9. When comparing batch fermentation runs in a laboratory fermentor and shake flask, it was noted that both resulted in comparable maximum concentrations of CPC, reaching 399.52 µg/mL and 424.2 µg/mL, respectively on day 4. However, a notable difference was observed in the time course of CPC production as shown in Fig. 10.

### Reverse phase HPLC analysis

As depicted in Fig. S2, the HPLC analysis demonstrated that the CPC peak of *A. chrysogenum* W42-I appeared at the identical retention time as the standard CPC (Fig. S2, supplementary file). The concentrations of CPC obtained from the corresponding inhibition zones were closely matched with those derived from the HPLC results as shown in Table S1 (supplementary file).

### Statistical analysis

Statistical analysis was calculated as mean ± SD using the Excel Microsoft Office 365.

## Discussion

As previously reported, the arthrospores formation of *A. chrysogenum* can be promoted by regulating the culture environment, ultimately boosting the production yield of fermentation products (Shahidzadeh et al. 2017). The CPC and its semi-synthetic variations, such as 7-aminocephalosporanic acid (7-ACA), play a crucial role as β-lactam antibiotics and are widely utilized (Lin and Kück 2022). These cephalosporins exhibit lower toxicity and a broader spectrum of antibiotic activity compared to ampicillin (Lin and Kück 2022). Various *Streptomyces* spp. and filamentous fungi are capable of producing cephalosporins, with *A. chrysogenum* being particularly notable for its extensive use in industrial CPC production, thanks to its high yield of CPC (Duan et al. 2014). In this research, our goal was to identify the best conditions for increasing the production of CPC on a larger scale. We investigated different environmental variables, including inoculum size, aeration, agitation rate, and pH, using the optimized media known as CPC2 from our previous study (Ibrahim et al. 2023). In every experiment, samples were collected at various time points to track the CPC generation. The effectiveness of CPC as an antibacterial agent was then evaluated through a bioassay conducted against standard *S. aureus*, ATCC 25,923, at the end of each trial (Bonev et al. 2008).

Following the optimization of culture media and production conditions at the shake flask level, as detailed in our previous study by Ibrahim et al. ( 2023), the subsequent phase involved scaling up to a laboratory fermentor. In fermentors, achieving higher cell concentrations than those attained in shake flasks is possible, primarily due to enhanced oxygen supply facilitated by air sparging and stirring. Moreover, fermentor settings, including dissolved oxygen (DO) and pH levels, can be monitored and regulated, contributing to improved CPC production (Villen et al. 2009). Nevertheless, it’s important to note that the outcomes derived from various experiments conducted at the shake flask level should be viewed solely as preliminary indicators for the optimal conditions necessary for scaling up to a fermentor.

After carrying out a run on the shake flask level, several fermentation runs were carried out in the present study, in a 14 L laboratory fermentor with the aim of determining the optimum fermentation conditions for maximum CPC production as a prerequisite step for industrial scaling up. All fermentation runs were carried out at a constant temperature of 28 °C and samples were withdrawn at specific time intervals for monitoring CPC concentration. First, batch fermentation was conducted using the optimized conditions obtained on the shake flask level using RSM (inoculum size of 1% v/v and initial pH of 4) to specify the time required for maximum CPC production. Note that pH was adjusted to 4 as a start, agitation rate of 200 rpm and an aeration rate of 0.5 vvm.

The clumping effect observed in *Acremonium* species fermentation involves the aggregation of fungal cells during the fermentation process, influencing various aspects of production (Sandor et al. 2001). Furthermore, the high viscosity of the culture growth is one of the characteristic traits of this strain resulting in an increased cost of energy to maintain the required level of the DO% (Almeida et al. 2001). Consequently, this was the primary reason for terminating the fermentation run after only six days.

The effect of the growth conditions on the synthesis of CPC was previously studied in several strains of *A. chrysogenum* (Bartoshevich et al. 1983, 1990; Karaffa et al. 1996). When different aeration rates were tested in this study, results revealed that 1 vvm gave a higher CPC concentration than that of 0.5 and 2 vvm. The CPC production is increased not only by supplementing oxygen but also by increasing the rate of agitation (Tollnick et al. 2004). Agitation rate is highly correlated with oxygen transfer efficiency which is a key factor for the oxidation of substrates in aerobic fermentation. Therefore, a suitable oxygen transfer rate can be achieved by means of agitation, which consequently improves the CPC yield. In the present study, experiments in the laboratory fermentor proved that agitation speed had a significant effect on CPC production and that increasing the agitation speed to 400 rpm led to a higher CPC production.

The control of microorganism activities within a specific system is greatly influenced by population density, also known as inoculum size. The growth of microorganisms and the manufacture of secondary metabolites are heavily dependent on the size and age of the inoculum. Furthermore, the biological state of the inoculum at the time it is moved to the production significantly affects the effectiveness of fermentation in the production of secondary metabolites (Gohar et al. 2019). Results showed that a smaller inoculum size of 1% v/v resulted in a higher maximum CPC concentration. A notable density of spores has the capacity to trigger rapid and extremely abundant biomass generation, thereby causing the swift exhaustion of nutrients and, ultimately, a reduction in the final product (Krishnaveni et al. 2009). In our study, increasing inoculum size to 2.5 & 5% v/v resulted in lower maximum CPC concentration. This phenomenon may be attributed to the intensified competition among bacteria when the size of the initial inoculum exceeded a particular threshold, thus causing a shift in metabolic patterns toward a more survival-oriented strategy (Bertrand 2019).

After selecting aeration rate of 1% vvm, agitation speed of 400 rpm & an inoculum size of 1% v/v, controlled pH was tested. Numerous fungi exhibit growth across a broad pH spectrum and their gene expression is adapted to environmental pH conditions (Peñalva et al. 2008; Prasetyo et al. 2011). Our strain demonstrated optimal CPC production at a pH of 4 which is a key finding that significantly influenced the fermentation process (Ibrahim et al. 2023). The importance of pH control became evident during Run 1, where the uncontrolled pH resulted in substantial fluctuations throughout the run. This highlighted the necessity of implementing pH control for achieving consistent and efficient CPC production by the tested isolate. Fortunately, with the implementation of controlled pH, the CPC production experienced a significant 1.4-fold increase. This underscores the positive impact of pH control in enhancing the efficiency of CPC production in the fermentation process.

Indeed, during large-scale operations, factors such as mixing time, gas distribution, nutrient concentration, and temperature circulation may not accurately replicate those observed at small scale. These variations have the potential to impact product pattern or transport within microbial cells. Consequently, it is wise to thoroughly evaluate the production during the process of scaling up to ensure consistency and meet desired outcomes (Baltz et al. 2010). The fundamental objective of scaling up a fermentation process is to validate the production of fermented products at a larger scale while maintaining consistent productivity and quality, mirroring the outcomes achieved during the initial small-scale development. Fortunately, this study achieved not only consistent production but also demonstrated improved production kinetics.

The utilization of a bioreactor for the fermentation process of *Acremonium chrysogenum* has been extensively investigated in numerous studies, leading to the achievement of a CPC yield of 0.315 g/L (Tabaraie et al. 2012) and 0.6 g/L (Nigam et al. 2007). These findings are comparable with our own results, where the maximum production of CPC resulted in a yield of 0.399 g/L.

HPLC analysis plays a crucial role in biotechnology and industrial microbiology. It allows for the detection and identification of trace levels of microorganisms’ byproducts (Fox et al. 2013). HPLC is known for its rapidity, specificity, accuracy, precision, and ease of automation, making it a widely used analytical technique (Upreti et al. 2024). One of the main advantages of HPLC is its ability to analyze multiple components in complex matrices, such as fermentation broths (Akmal et al. 2000). HPLC also provides excellent long-term stability, making it suitable for automation and true online monitoring in bioproduction processes (Compton 1990). In our study, the accuracy of the data was confirmed through HPLC analysis and the results from both HPLC and agar well diffusion methods were consistent with each other.

Many researchers have undergone computational analysis aimed for protein engineering of the key enzymes such as CPC acylase (Deshpande et al. 1996; Tian et al. 2017b; Pollegioni et al. 2013) and thioredoxin reductase (Liu et al. 2013) which are involved in the biosynthesis of CPC to optimize and stabilize the produced CPC. Therefore, our future perspective is to act on certain enzymes that previously confirmed to be involved in the biosynthesis (Tian et al. 2017b; Pollegioni et al. 2013; Liu et al. 2013) or in the modification of CPC (Takimoto et al. 2004; Tian et al. 2014). This has to be done in order to not only optimize CPC production but also to synthesis new derivatives of CPC to act as precursor of newly synthesized cephalosporins. Previous reports of homologous expression of certain genes have been conducted using *A. cellulolyticus* strain and were successfully employed to optimize production of valuable industrial enzymes significantly such as xylanase (Watanabe et al. 2014), cellulase, hemicellulose (Fujii et al. 2023) and β-xylosidase (Kanna et al. 2011). Moreover, applying qPCR analysis of *Acremonium chrysogenum* strains with CPC production differences greater than 100-fold, the transcript levels of numerous important genes involved in CPC biosynthesis and transport were previously identified (Dumina et al. 2014). The wild-type ATCC 11,550 strain was much less productive than the high-producing RNCM F-4081D strain when it came to the expression of genes involved in the final phases of CPC manufacture (Dumina et al. 2014). In conclusion, our findings of this study showed that the best conditions for producing CPC in a laboratory fermentor were an inoculum size of 1% v/v, aeration rate of 1 vvm, agitation rate of 400 rpm, and maintaining a controlled pH of 4 reaching a concentration of 399.52 µg/mL. This showed about 3.44-fold increase when compared to the unoptimized fermentation run (Run 1). Moreover, our results revealed that the time course for CPC production in the laboratory fermentor was more favorable compared to that previously obtained using the shake flask (39.89 µg/mL). Remarkably, there was a twofold increase in production within the initial three days followed by a constant production for three successive days. The fermentor succeeded in scaling up CPC production to reach a yield of 1.598 gm/ 4 L. However, further studies are highly recommended to further optimize CPC production by using genetically modified or pathway-engineered *A. chrysogenum* strains. This can be carried out through gene duplication, homologous or heterologous expression of the key genes whose gene products are confirmed to be involved in the biosynthetic pathway of CPC.

## Electronic supplementary material

Below is the link to the electronic supplementary material.

Supplementary Material 1

## Data Availability

and Material. All data generated or analyzed during this study are included in this published article and supplementary file.
