# Supplementary material for: Scaling up production of cephalosporin C by Acremonium chrysogenum W42-I in a fermenter using submerged fermentation
Source: AMB Express. 2024 Nov 5;14:121. doi: 10.1186/s13568-024-01778-1 (PMC11538202; doi:10.1186/s13568-024-01778-1)
Supplement: Supplementary file 1 — Supplementary Material 1 [file 13568_2024_1778_MOESM1_ESM.docx]

**-Journal name**: AMB Express

**-Manuscript Title**: Scaling up production of cephalosporin C by *Acremonium chrysogenum* W42-I in a fermenter using submerged fermentation

Asmaa A. Ibrahim^1^, Ghadir S. El-Housseiny^1^, Khaled M. Aboshanab*^1^, Ansgar Stratmann^2^_,_ Mahmoud A. Yassien^1^, Nadia A. Hassouna^1^,

^1^Department of Microbiology and Immunology, Faculty of Pharmacy, Ain Shams University, Cairo 11566, Egypt

^2^W42 Industrial Biotechnology GmbH, 44227 Dortmund, Germany. [a.stratmann@w42biotechnology.de](mailto:a.stratmann@w42biotechnology.de)

* Corresponding Author: Prof. Dr Khaled Aboshanab, PhD

Address: Department of Microbiology and Immunology, Faculty of Pharmacy, Ain Shams University, Organization of African Unity St., POB: 11566, Abbassia, Cairo, Egypt.

E-mail:[aboshanab2012@pharma.asu.edu.eg](mailto:aboshanab2012@pharma.asu.edu.eg)

ORCID: <https://orchid.org/0000-0002-7608-850X>

Tel: (202)28434595

Mobile: 01007582620

Fax: (202)24051107

E-mails of Coauthors:

Asmaa A. Ibrahim: [asmaa.zaki@pharma.asu.edu.eg](mailto:asmaa.zaki@pharma.asu.edu.eg)

Ghadir S. El-Housseiny: [ghadir.elhossaieny@pharma.asu.edu.eg](mailto:ghadir.elhossaieny@pharma.asu.edu.eg)

Ansgar Stratmann: [a.stratmann@w42biotechnology.de](mailto:a.stratmann@w42biotechnology.de)

Mahmoud A. Yassien: [mahmoud.yassien@pharma.asu.edu.eg](mailto:mahmoud.yassien@pharma.asu.edu.eg)

Nadia A. Hassouna: [nadia.hassouna@pharma.asu.edu.eg](mailto:nadia.hassouna@pharma.asu.edu.eg)

**
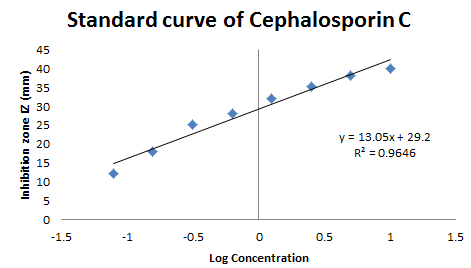
**

**Fig**. S**1** Standard Curve of standard CPC antibacterial activity against *S. aureus* ATCC 25923


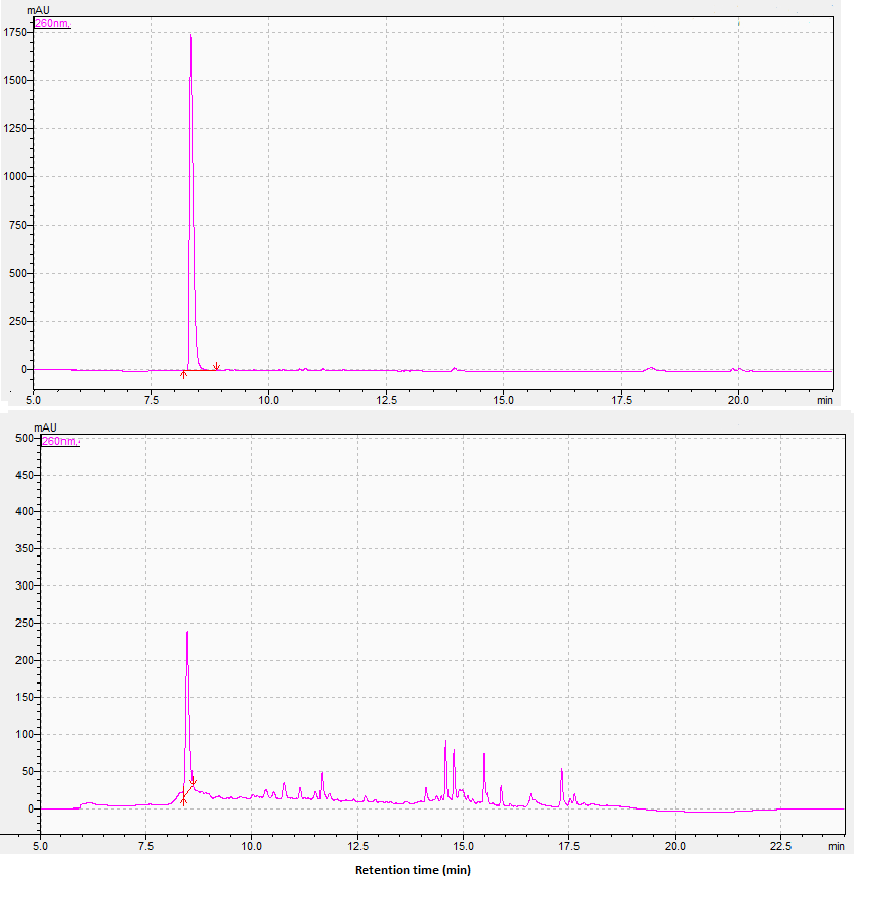


**Figure S2**. HPLC Chromatograms of: A: Standard CPC; B: Chromatogram of the CPC produced by A. chrysogenum W42-I.

**Table S1.** Summary of maximum CPC production in different studied batch fermentation runs.

| **Run no.** | **Conditions** | | **IZ (mm)** | **CPC conc.**  **(mg/mL)** | **Approximated AUC**  ***10ˆ5** | **CPC conc. (mg/ml)** | **Fermentation day where Max. CPC reached** |
| --- | --- | --- | --- | --- | --- | --- | --- |
|  | Varied | Common |  |  |  |  |  |
| 1 | Aeration rate 0.5 vvm | Initial pH 4, agitation 200 rpm, Inoculum size 1% v/v, temperature 28°C | 17 | 0.11 | 10 | 0.1 | 6 |
| 2 | Aeration rate 1 vvm |  | 20 | 0.19 | 18 | 0.18 | 5 |
| 3 | Aeration rate 2 vvm |  | 19 | 0.16 | 15 | 0.156 | 5 |
| 4 | Agitation rate 300 rpm | Aeration rate 1 vvm Inoculum size 1% v/v, initial pH 4, temperature 28°C | 21 | 0.23 | 24 | 0.235 | 6 |
| 5 | Agitation rate 400 rpm |  | 22 | 0.28 | 27 | 0.277 | 4 |
| 6 | Inoculum size 2.5% v/v | Agitation rate 400 rpm, aeration 1 vvm, initial pH 4, temperature 28°C | 18 | 0.13 | 10.98 | 0.11 | 6 |
| 7 | Inoculum size 5% v/v |  | 20 | 0.19 | 18.48 | 0.185 | 6 |
| 8 | Controlled pH 4 | Inoculum size 1% v/v, agitation rate 400 rpm, aeration 1 vvm, temperature 28°C | 24 | 0.39 | 37.96 | 0.38 | 4 |

AUC, area under the curve; CPC, cephalosporin C; IZ, inhibition zone.
